# An unusual case of perforated stump appendicitis: A case report

**DOI:** 10.1016/j.amsu.2022.103447

**Published:** 2022-03-09

**Authors:** Sunil Basukala, Bishnu Deep Pathak, Soumya Pahari, Suman Gurung, Bikram Basukala, Bikash Bahadur Rayamajhi, Narayan Thapa

**Affiliations:** a Department of Surgery, Shree Birendra Hospital, Chhauni, Kathmandu, 44600, Nepal; b Nepalese Army Institute of Health Sciences (NAIHS), Sanobharyang, 44600, Kathmandu, Nepal; c Department of Pathology, Shree Birendra Hospital, Chhauni, Kathmandu, 44600, Nepal

**Keywords:** Stump appendicitis, Abdominal pain, Right iliac fossa, Septic shock

## Abstract

**Introduction and importance:**

The stump appendicitis is a rare complication with incidence of 1 in 50,000 appendectomy cases.

**Case presentation:**

Patient with a history of emergency open appendectomy one year back presented with symptoms as that of acute appendicitis like pain abdomen localized in right iliac fossa, nausea, vomiting and anorexia. Complete blood count showed leukocytosis.

**Clinical discussion:**

Aside from classical clinical symptoms similar to acute appendicitis other causes of acute abdominal pain were ruled out with clinical laboratory and radiological investigations. This creates a dilemma and delay in diagnosis if investigations are not done promptly.

**Conclusion:**

Due to prior surgical history of appendectomy and low index of suspicion, the diagnosis of stump appendicitis is often delayed which may result in serious complications like stump gangrene, perforation and peritonitis.

## Highlights

• Stump appendicitis is one of the rare complications of appendectomy.
• Stump appendicitis can occur mainly in patients who have undergone either laparoscopic or open appendectomy and may pose a serious diagnostic dilemma.
• Surgical resection (appendectomy) either open or laparoscopic is the most appropriate treatment in these cases.
• The interval between the initial appendectomy and the new presentation can range from 4 days to 50 years.

## 1 Introduction

Appendectomy is one of the most commonly performed surgical procedures worldwide [1]. The stump appendicitis is a rare complication with an incidence of 1 in 50,000 appendectomy cases [2]. It is characterized by obstruction and inflammation of residual appendiceal tissue after appendectomy [3]. It is said that this complication develops more often after laparoscopic surgery due to incomplete resection of appendix. It may occur at variable time after initial surgery, ranging from two months to 50 years [4]. Patients present with symptoms such as that of acute appendicitis like pain abdomen, nausea, vomiting and anorexia. Complete blood count may show leukocytosis. This creates a dilemma and delay in diagnosis [1]. The rate of perforation has been reported to be around 70% [4] Computerized Tomography of Abdomen is the most commonly used diagnostic modality [5]. Completion appendectomy is the treatment of choice [6,7]. This case report is being reported in line with the SCARE 2020 criteria [8].

## 2 Method

We reported this case following the updated consensus-based Surgical Case Report (SCARE) guidelines [8].

## 3 Case presentation

A 28-year-old male presented to the Emergency Room with complaints of right lower abdominal pain for 12 hours, which was insidious in onset, continuous, non-radiating, dull aching type with no exacerbating and relieving factors. It was associated with nausea and anorexia as well. His past surgical history included open appendectomy for acute appendicitis one year back with an uneventful postoperative period. No current medication use, family history was insignificant and the patient is a non-smoker. The patient was afebrile and the vitals were within normal limits. Abdominal examination showed scar of previous gridiron incision. There was tenderness and rebound tenderness in the right iliac fossa. Mild guarding was present with no other peritoneal signs.

**Fig. 1:**
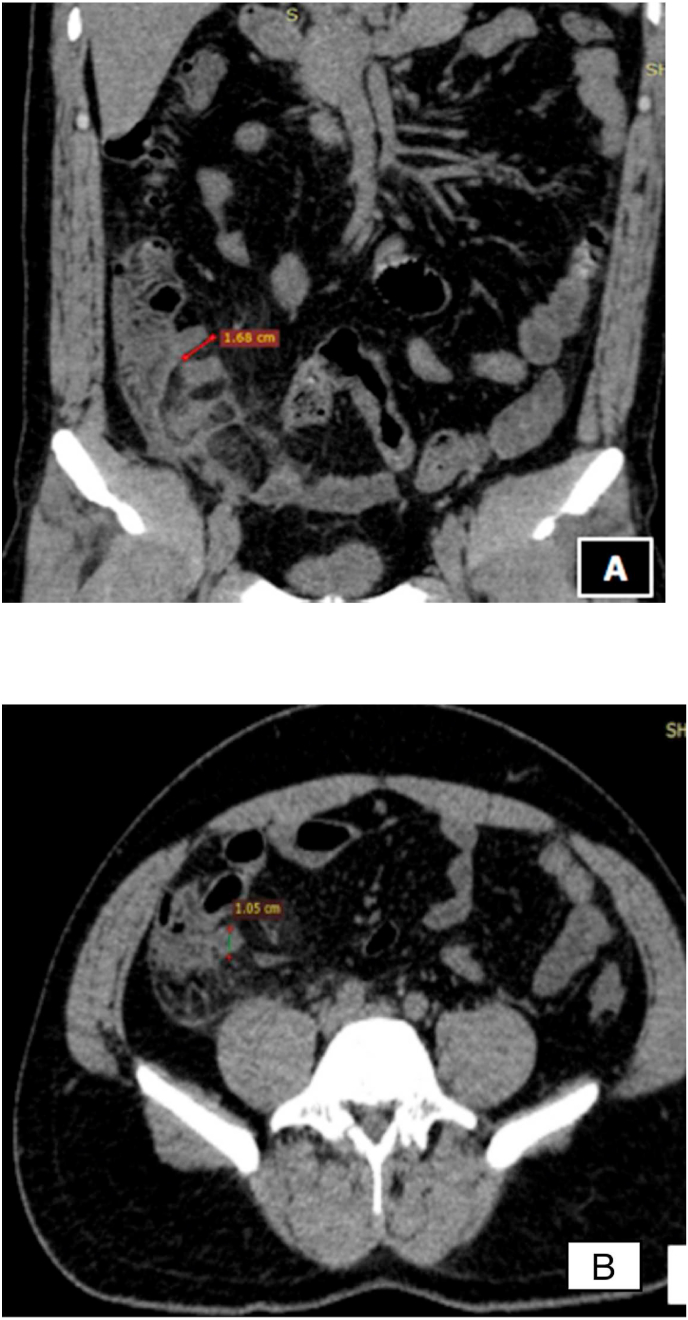
Contrast Enhanced Computerized Tomography (CECT) of abdomen and pelvis A. Coronal Section B. Axial section showing stump appendix (Arrow indicating appendiceal stump with inflammatory changes).

The routine laboratory tests were unremarkable except for leukocytosis of 14,500 cells per cubic mm with neutrophilic predominance (90%). Transabdominal ultrasonography showed evidence of fluid collection in the right paracolic gutter with no other abnormalities. However, Contrast Enhanced Computerized Tomography (CECT) of abdomen and pelvis revealed a small structure at the base of caecum with enhancing walls along with stranded surrounding fat, suggestive of inflamed appendiceal stump. This confirmed the diagnosis of stump appendicitis Fig. 1.

**Fig. 2:**
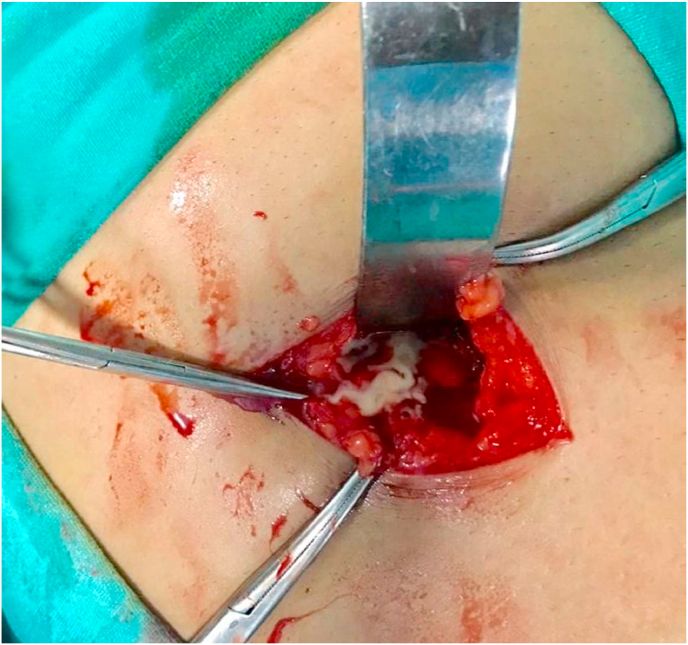
Intraoperative abscess on exploration of perforated stump appendix.

The patient was immediately shifted to the operation room. A consultant general surgeon with prior experience performed the surgery. Abdomen was opened via gridiron incision. The paracolic gutter was packed with purulent fluid. The caecum and ascending colon were mobilized, and taenia coli were traced up to the base of the appendiceal stump. There was evidence of perforated abscess at the site of the stump. The purulent fluid (Fig. 2.) was drained and the residual appendiceal tissue was resected.

**Fig. 3:**
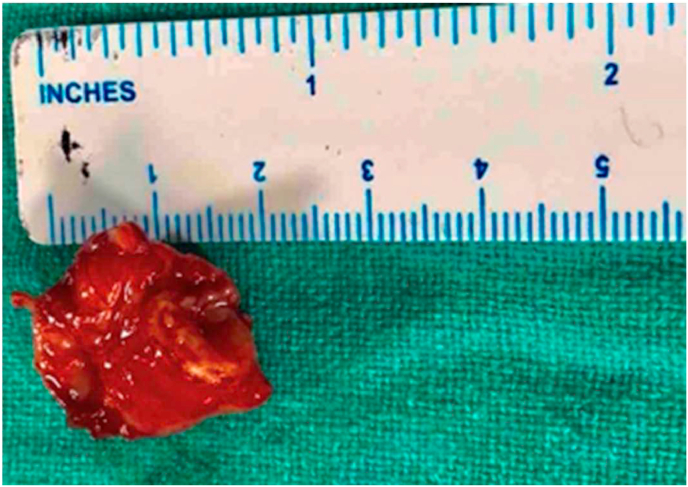
Gross specimen of Stump appendix postoperatively.

**Fig. 4:**
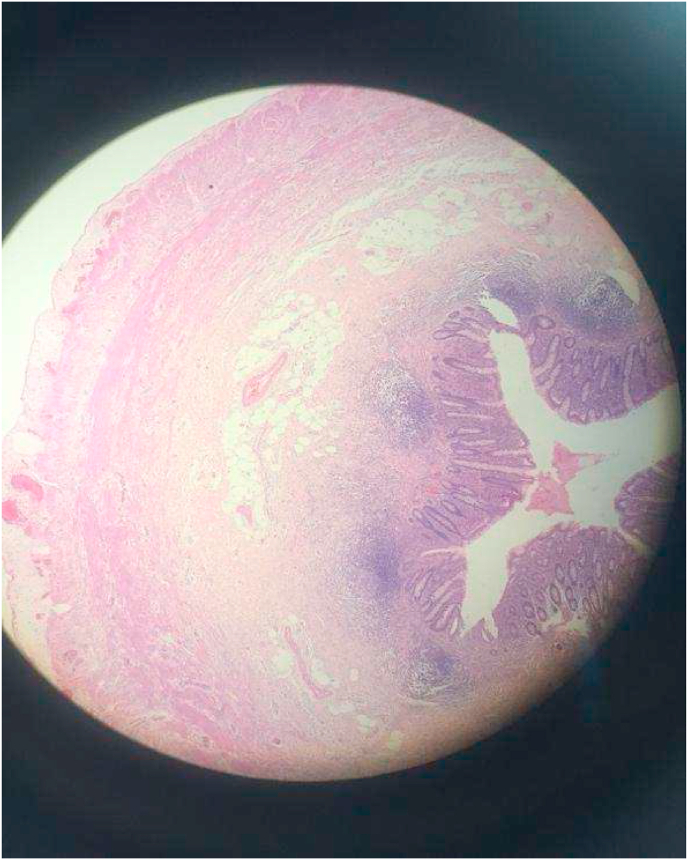
H&E section of Appendix (40X & 100X).

The peritoneal cavity was thoroughly washed with normal saline. The tissue sample was sent for histopathological examination Fig. 3 which confirmed the diagnosis of appendicitis, making the final diagnosis as perforated stump appendicitis. Fig. 4.

Following surgery, the patient was transferred to post-operative room. He was put on parenteral antibiotics and analgesics. He did well during the postoperative period, and was discharged on day five. On follow up after two weeks, the patient was stable and healthy.

## 4 Clinical Discussion

Stump appendicitis is a well-recognized entity [1] though its occurrence is rare [9] with incidence being 1 in 50,000 appendectomies. It is defined as the interval development of obstruction and inflammation of any remaining appendix after an appendectomy [10]. Due to prior surgical history of appendectomy and low index of suspicion, the diagnosis of stump appendicitis is often delayed which may result in serious complications like stump gangrene, perforation and peritonitis [9]. Rate of perforation, which was also seen in our case, is extremely high, approaching 70% [11]. A literature review by R. Hendahewa et al. showed that SA was frequently misdiagnosed as constipation, gastroenteritis or right sided diverticulitis, therefore leading to a significant delay to surgery [2].

The age of patients presenting with stump appendicitis ranges from 8 to 80 years and male to female ratio is 1.1:1 [4]. It may present as acute or subacute appendicitis. It is recognized that long appendiceal stump and improper identification of stump during appendectomy is the main risk factor for residual appendix [9]. The risk factors are divided into anatomical and surgical related factors. Inappropriate identification of appendiceal base or appendico-cecal junction is a common denominator to both. Retrocecal appendix or subserosal appendix, a duplicated appendix or diverticulum at the base of appendix are some of the contributing anatomical factors. Surgery related factors include inadequate identification of the appendicular base because of severe local inflammation, leaving a long stump due to fear of cecal injury or difficult dissection and local ulcerations due to presence of a faecolith. Both of the surgical techniques for dealing with the stump i.e., inversion of stump or simple ligation of stump do not prevent the possibility of stump appendicitis [2]. Increase in incidence of SA has been attributed to introduction of laparoscopic appendectomy [9]. Lack of three-dimensional vision and absence of tactile feedback in laparoscopy has been suggested by some authors to increase the chance of leaving behind a longer stump [2]. However, a review by Liang et al. revealed that incidence of stump appendicitis after standardized laparoscopic appendectomy is less than half as compared to open technique [[9], [10], [11]]. The incidence should not be increased if laparoscopic appendectomy is performed properly [1].

A review of 61 cases by A. Subramanian and M.K. Lang showed mean stump length of 3.3 cm and appendiceal stumps of less than 0.5 cm are unlikely to result in future stump appendicitis [10]. Mangi and Berger [10] have suggested that the incidence of stump appendicitis can be decreased by proper identification of the base of the appendix and by leaving an appendiceal stump of <3 mm long. Dissecting the recurrent branch of the appendiceal artery and following the tenia coli on the caecum helps in identifying the true appendicular base.

High index of clinical suspicion supported by investigations such as abdominal ultrasound scan or 10.13039/100004811CT scan aid in the diagnosis of Stump Appendix [[11], [12], [13], [14], [15]]. CT scan excludes other etiologies of the acute abdomen and it should be able to identify pericecal inflammatory changes, abscess formation, fluid in the right paracolic gutter, cecal wall thickening, ileocecal mass and, in some cases, a prompt visualization of the appendicular stump can be made, hence it is preferred over ultrasound scan [15]. Diagnostic laparoscopy is useful in some doubtful cases with persistent abdominal symptoms after ruling out other pathologies by doing extensive imaging and It may allow safe completion appendectomy if diagnosis of stump appendicitis is made. Colonoscopy and barium enema are other modalities of diagnosis described in the literature [16].

The completion appendectomy is the treatment of choice for stump appendicitis, most commonly done as an open operation (72%) [15] but a successful laparoscopic approach has also been reported. Only 10% of stump appendicitis which are perforated have been treated with laparoscopic procedure, the rest requiring open surgery [11]. Extensive procedures like ileocecostomy may be required in the setting of extensive inflammation and peritonitis [1]. Perforated stump appendicitis requiring partial cecectomy and right hemicolectomy have also been reported [11]. Non-surgical treatments have also been described for stump appendicitis, which include colonoscopic removal of appendicolith [[15], [16], [17]] and medical management using antibiotics, analgesics and clear liquid diet. Such patients may undergo interval stump appendectomy after 6 weeks, following resolution of inflammation. This is said to decrease the potential morbidity of wound and surgical site infections and eliminate repeated bouts of stump appendicitis in the future [16,17].

## 5 Conclusion

Stump appendicitis can represent a diagnostic dilemma if the treating physician is unfamiliar with this uncommon clinical entity. The timely use of CT scan allows the prompt diagnosis in cases with perforated sump appendicitis. Completion appendectomy either open or laparoscopic method is the definitive modality of treatment.

The primary takeaway lesson from this case report is that the treating physician/surgeon should have a high index of suspicion for this rare entity while dealing with a patient having abdominal pain and a past surgical history of appendectomy, in order to establish a timely diagnosis and avoid potential complications that might follow.

## Ethical approval

The case report is exempt from ethical approval in our institution.

## Sources of funding

This research did not receive any specific grant or funding from funding agencies in the public, commercial, or not-for-profit sectors.

## Author contribution

Sunil Basukala (SB) = Conceptualization, Supervision.

Sunil Basukala (SB), Bishnu deep Pathak (BDP), Soumya Pahari (SP) = Writing - original draft.

Sunil Basukala (SB), Bikash Bahadur Rayamajhi (BBR), Bikram Basukala (BB), Suman Gurung (SG) = Writing - review & editing.

All the authors read and approved the final manuscript.

## Consent

Written informed consent was obtained from the patient for publication of this case report and accompanying images. A copy of the written consent is available for review by the Editor-in-Chief of this journal on request.

## Research Registration Number

Not applicable.

## Guarantor

Sunil Basukala (SB).

## Provenance and peer review

Not commissioned, externally peer-reviewed.

## Declaration of competing interest

All authors declare that they have no conflict of interest.
